# Investigation of L-Tryptophan Electrochemical Oxidation with a Graphene-Modified Electrode

**DOI:** 10.3390/bios11020036

**Published:** 2021-01-28

**Authors:** Florina Pogacean, Codruta Varodi, Maria Coros, Irina Kacso, Teodora Radu, Bogdan Ionut Cozar, Valentin Mirel, Stela Pruneanu

**Affiliations:** National Institute for Research and Development of Isotopic and Molecular Technologies, Donat Street, No. 67-103, 400293 Cluj-Napoca, Romania; florina.pogacean@itim-cj.ro (F.P.); codruta.varodi@itim-cj.ro (C.V.); maria.coros@itim-cj.ro (M.C.); irina.kacso@itim-cj.ro (I.K.); teodora.radu@itim-cj.ro (T.R.); bogdan.cozar@itim-cj.ro (B.I.C.); valentin.mirel@itim-cj.ro (V.M.)

**Keywords:** electrochemical exfoliation, doped graphene, heteroatoms, enhanced detection

## Abstract

A graphene sample (EGr) was prepared by electrochemical exfoliation of graphite rods in solution containing 0.05 M (NH_4_)_2_SO_4_ + 0.1 M H_3_BO_3_ + 0.05 M NaCl. The exfoliation was performed by applying a constant voltage (12 V) between the graphite rods, while the temperature was kept constant (18 °C) with a temperature-controlled cryostat. The structural investigation of the graphene sample, performed by X-ray powder diffraction (XRD), revealed that the sample consists of a mixture of few-layer (69%), multi-layer graphene (14%) and graphene oxide (17%). In addition, XPS analysis proved that the sample was triple-doped with heteroatoms such as nitrogen (1.7 at%), sulfur (2.5 at%), and boron (3 at%). The sample was deposited onto the surface of a clean, glassy carbon electrode (GC) and investigated for the non-enzymatic electrochemical detection of L-tryptophan (TRP). The electrocatalytic properties of the EGr/GC electrode led to a considerable decrease in the oxidation potential from +0.9 V (bare GC) to +0.72 V. In addition, the EGr/GC electrode has higher sensitivity (two times) and a lower detection limit (ten times) in comparison with the bare GC electrode.

## 1. Introduction

In the last few years, intensive research has been devoted to the development of analytical methods useful for the detection of biomolecules present in living species. Since various biomolecules coexist in the human body, it is a difficult task to detect them in a single run due to their different physical–chemical properties. The nonenzymatic electrochemical methods may be useful for the detection of important biomolecules (e.g., L-tryptophan, ascorbic acid, dopamine, uric acid) based on the fact that they have different oxidation potentials.

To fulfill the increasing interest in ultrasensitive detection, scientists have developed novel procedures to improve the response of electrochemical sensors by modifying them with various functional materials [1]. Due to its diverse structural, morphological, and chemical properties, graphene has found applications in numerous fields, including sensing material [2]. Nevertheless, pristine graphene tends to agglomerate, causing a loss of performance in the detection process. Therefore, it is necessary to modify graphene in order to diminish this weakness. Doping graphene with nitrogen, boron, sulfur, phosphorus, or combinations of these elements can substantially improve the characteristics of the materials [3]. So far, a large number of reports have confirmed the positive effects of heteroatom doping on the electrochemical property enhancement of graphene [4]. Multiple doping is more efficient in comparison with mono-heteroatom doping since co-doped graphene materials have revealed a range of unique properties due to the synergistic effect of multiple-element doping [5]. Heteroatom doping of graphene can be achieved by the CVD method [6], thermal annealing of graphene oxide (GO) with heteroatom precursors [7], plasma [8] and arc-discharge [9] techniques, or solvothermal synthesis [10]. Recently, electrochemical exfoliation of graphite has attracted attention due to its easy, rapid, and environmentally friendly approach to producing high-quality doped graphene [11]. Although co-doped carbon materials potentially possess promising properties for various applications, there are only a few reports about graphene that is simultaneously doped with nitrogen, sulfur, and boron [12]. 

Tryptophan is an essential amino acid for humans [13] and a precursor for the neurotransmitter serotonin or the hormone melatonin [14], important substances in the modulation of several important behaviors and psychological functions such as sleep, emotional state, perception, and circadian rhythm [15]. A significant association between plasma tryptophan concentrations, sleep, and good mental health has been reported [16]. Consequently, a sensitive method for tryptophan determination is very useful. Among the methods used for the determination of tryptophan, chromatographic [17], chemiluminescence [18], capillary electrophoresis [19], and electrochemical techniques [20] were highly sensitive, accurate, and simple to use. Various materials for electrode modification have been employed so far, such as nitrogen-doped graphene nanosheets/CuCo_2_O_4_ [21], poly(L-methionine)/graphene [22], a flower-like-structured nanocomposite consisting of reduced graphene oxide and SnO_2_ [23], Cu_2_O-nanoparticle-coated reduced graphene oxide [24], polyvinylpyrrolidone-functionalized graphene [25], and 3D nitrogen and sulfur co-doped graphene/integrated polysaccharides [26]. To the best of our knowledge, no study has been published on tryptophan determination using nitrogen-, sulfur-, and boron-doped graphene. 

In this work, we report an easy electrochemical method for the synthesis of good-quality triple-doped graphene (N, S, B) in aqueous electrolytes containing ammonium sulfate, boric acid, and sodium chloride. Our method has the advantage of short processing time and mild reaction conditions. Incorporating N, S, B into the electrochemically exfoliated graphene sheets showed favorable electrocatalytic activity towards tryptophan detection. 

## 2. Materials and Methods

### 2.1. Instruments

An SEM/TEM Hitachi HD2700 instrument (Hitachi, Japan), equipped with a cold field emission gun (CSEG), operated at 200 kV, and coupled with a double-cut windowless 100 EDX detector using AZtec Software (Oxford Instruments, Oxford, UK), was used for the morphological characterization of the graphene sample.

The X-ray powder diffraction (XRD) pattern was collected with a Bruker D8 Advance Diffractometer using CuK_α1_ radiation (λ = 1.5406 Å). Background-corrected patterns were used for the determination of some structural parameters. 

The FTIR spectrum measurement (4000–400 cm^−1^ spectral domain) was obtained with a resolution of 4 cm^−1^ using a JASCO 6100 FTIR spectrometer (KBr pellet technique). Around 1 mg of graphene was mixed with ~200 mg KBr and grounded in an agate mortar. The grounded mixture was pressed into a pellet; then, the spectrum was immediately recorded.

The X-ray photoelectron spectroscopy (XPS) technique was employed to investigate the surface chemical composition of the sample. A SPECS spectrometer, equipped with an Al anode X-ray source and a PHOIBOS 150 2D CCD hemispherical energy analyzer, was used for the measurements. The X-ray source (Al K_α_, 1486.6 eV) was operated at 200 W. High-resolution X-ray photoelectron spectra for individual elements (C, O, B, S) were recorded by accumulating scans at constant pass energy of 30 and 0.1 eV/step. Data analysis and deconvolutions were performed using CasaXPS software with a Gaussian–Lorentzian product function and a nonlinear Shirley function for background subtraction. Peak shifts due to any apparent charging were calibrated by attributing the energy value of 284.6 eV to the C 1 s peak. 

A typical three-electrode cell, coupled with a potentiostat/galvanostat instrument (PGSTAT-302N, Metrohm-Autolab B.V., The Netherlands), was used for the electrochemical measurements (cyclic voltammetry (CV), linear sweep voltammetry (LSV), and amperometric measurements). The CV and LSV measurements were generally run between +0.2 and + 1 V vs. Ag/AgCl, with a scan rate of 10 mV·s^−1^. 

### 2.2. Chemicals

All chemicals, including ammonium sulfate (Reactivul Bucuresti), boric acid (Sigma-Aldrich, Sternheim, Germany), sodium chloride (Reactivul Bucuresti, Bucharest, Romania), potassium ferrocyanide (Sigma-Aldrich, Sternheim, Germany), potassium chloride (Sigma-Aldrich, Sternheim, Germany), L-tryptophan (Sigma-Aldrich, Sternheim, Germany), and dimethylformamide (DMF; JTBaker, HPLC grade, Sternheim, Germany) were used without further purification. Double-distilled water produced with Fistreem Cyclon equipment was employed to prepare all electrolyte solutions.

### 2.3. Synthesis of Graphene by Electrochemical Exfoliation of Graphite Rods (EGr)

**Figure 1:**
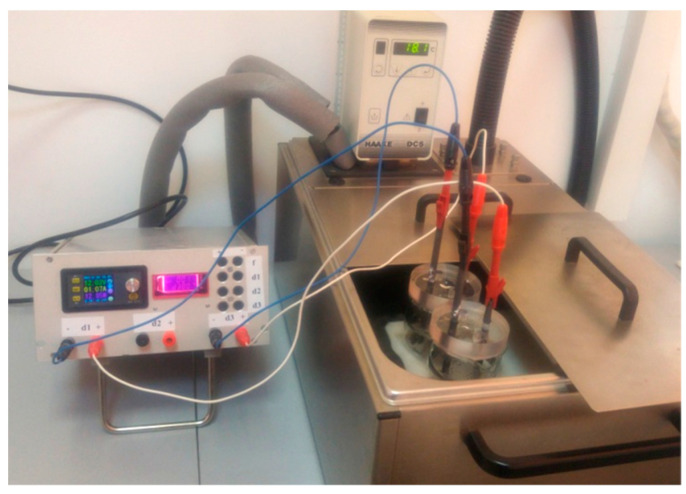
The experimental setup employed for the electrochemical exfoliation of graphite rods with pulses of current.

The graphene sample was prepared by electrochemical exfoliation of graphite rods, as described in the following: two electrochemical cells filled with solutions containing 0.05 M (NH_4_)_2_SO_4_ + 0.1 M H_3_BO_3_ + 0.05 M NaCl were connected to the exfoliation system. The exfoliation was performed by applying a constant voltage of 12 V between the graphite rods (anode and cathode), while the temperature was kept constant (18 °C) with a temperature-controlled cryostat (Figure 1). In order to minimize the heat generated in the reaction cells, short pulses of current were applied for a certain period of time (0.8 s), followed by short pauses (0.2 s). After 4 h, the material collected from the two cells was washed with distilled water (10 L) and then dispersed by ultrasound for 30 min in 125 mL water. Then, it was filtered on white-ribbon paper and dried by lyophilization. The sample was denoted as EGr.

### 2.4. Preparation of a Graphene-Modified Electrode (EGr/GC)

**Scheme 1:**
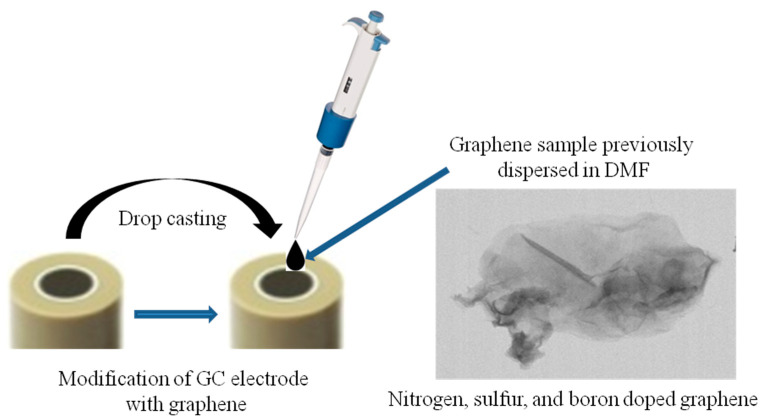
Schematic representation of glassy carbon electrode (GC) modification with the graphene sample previously dispersed in dimethylformamide (DMF).

The graphene sample previously dispersed in *N*,*N*-dimethylformamide (1 mg/mL) was sonicated for 3 min with a finger device (SONICS Vibra-Cell). Next, a volume of 10 µL from the graphene suspension was drop-casted onto a clean GC surface and dried at room temperature for 24 h. The schematic representation of GC modification with the EGr sample is shown in Scheme 1.

## 3. Results and Discussion

### 3.1. Morphological and Structural Characterization of the EGr Sample

**Figure 2:**
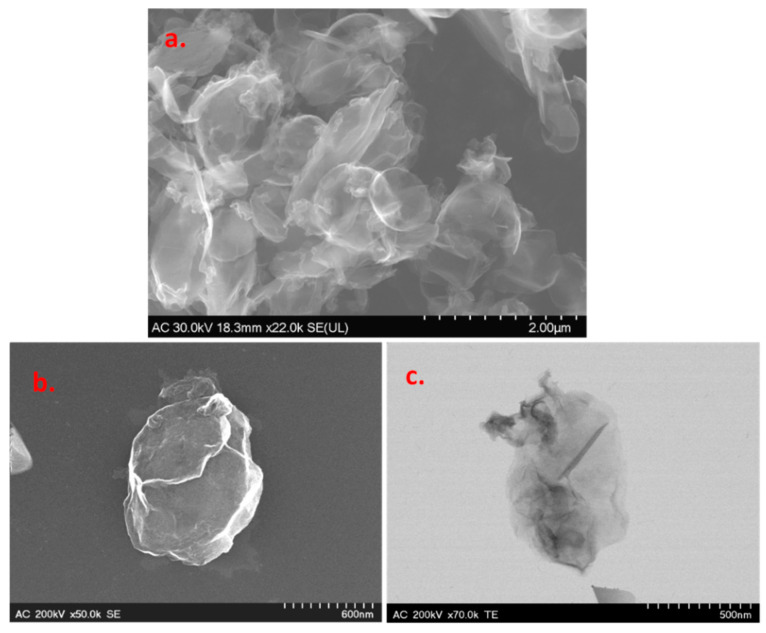
Representative SEM (**a**,**b**) and TEM (**c**) images of the graphene sample.

Scanning and transmission electron microscopy (SEM/TEM) techniques were employed for the morphological characterization of the graphene sample. Representative images are presented in Figure 2, where one can see smooth and large areas corresponding to the basal plane of graphene, along with sharp edges where there is graphene layer roll-up. The darker regions in the TEM image represent multilayer graphene, which is formed due to the strong π–π stacking interaction between single-layer graphene. 

**Figure 3:**
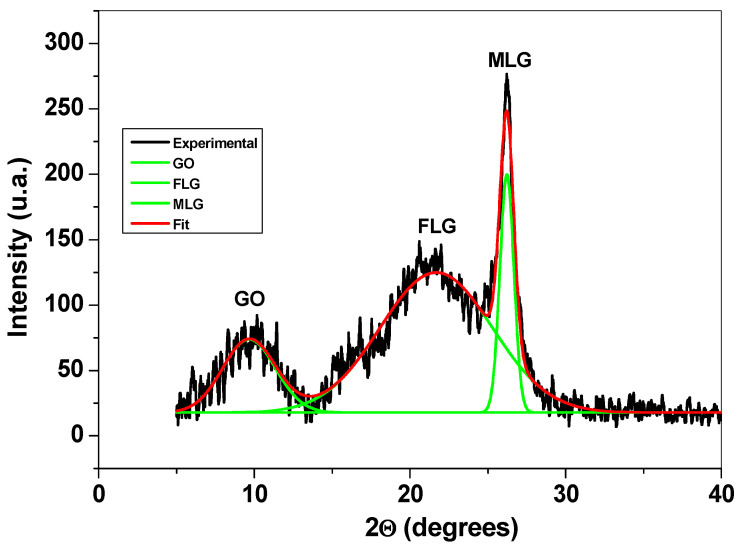
The XRD pattern of the graphene sample and the corresponding deconvoluted peaks.

Structural characterization of the graphene sample was next performed by XRD, FTIR, and XPS. Figure 3 presents the XRD pattern of the sample, where three main peaks can be observed: the first one is at around 10° and corresponds to the reflexions of graphene oxide (GO) flakes; the second and third peaks appear at around 21° and 26°, respectively, and are due to the reflexions of few-layer (FLG) and multi-layer graphene (MLG). The XRD technique was previously used to characterize graphene-based materials and to determine the average number of graphene layers (n), the interlayer spacing (d), the mean crystallite size (D), and the amount (%) of GO, FLG, and MLG within the sample [27,28,29].

For each peak, the *D* value was calculated from the full width at half maximum (FWHM) using the Debye–Scherrer equation (Equation (1)):

(1) $$D=\frac{0.9*\lambda }{\beta *\cos\theta }$$

where λ is the X-ray wavelength, β is FWHM expressed in radians, and θ is half the diffraction angle of the peak (in degrees), corresponding to interlayer distance, *d*. The *d* value was found with the Bragg equation (*d = λ/2 sin θ*), while the average number of layers, *n*, was calculated from the relation *n = D/d* [30].

The structural parameters obtained from the diffraction pattern are presented in Table 1. From this table, one can see that the sample contains a large amount of few-layer graphene (69%), along with smaller amounts of multi-layer graphene (14%) and graphene oxide (17%). 

**Figure 4:**
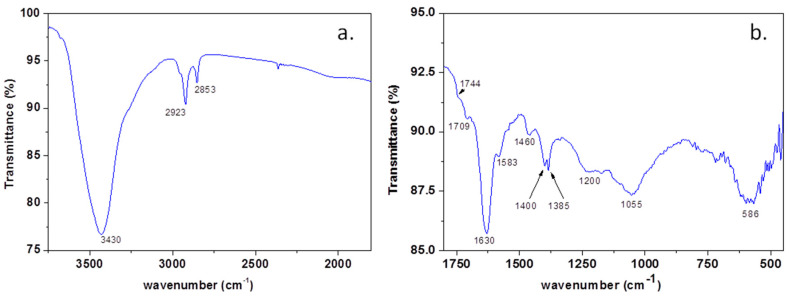
The FTIR spectrum of the graphene sample within 4000–2500 cm^−1^ (**a**) and 2000–450 cm^−1^ (**b**).

In order to prove the presence of heteroatoms within the graphene sample, the FTIR spectrum (Figure 4a,b) was recorded and interpreted, in good agreement with the literature data. The vibration bands identified in the FTIR spectrum of the sample can be assigned as follows: the broad band at 3430 cm^−1^ is due to -OH and NH_2_ stretching [31,32]; 2923 and 2853 cm^−1^ is assigned to asymmetric and symmetric stretching of -CH_2_ [33]; 1744sh and 1709 cm^−1^ is assigned to Ar-COOH stretching and C=O stretching [29,34]; 1630 cm^−1^ is assigned to -NH_2_ and aromatic C=C stretching; 1583 cm^−1^ is assigned to C=C and C=N stretching [31]; 1546sh cm^−1^ to C=C stretching [34], 1400 cm^−1^ to -COO^−^ stretching; 1384 cm^−1^ to C_ar_-N stretching and -OH deformation [31]. 

The broad band between 1276–1135 cm^−1^ is due to the overlapping of -OH bending and C-O-C and B-C stretching; 1100–950 cm^−1^, with maxima at 1055 cm^−1^ of -C-O and B-O-B stretching; 670–500 cm^−1^, with maxima at 586 cm^−1^ of O-B-O stretching [31,32,34].

The XPS investigation of the graphene sample further confirms the presence of heteroatoms within the sample (N, S, B). The chemical composition of the sample was estimated from the survey spectrum (data not shown), and the elemental concentrations are shown in Table 2. Here, one can see that S and B have similar concentrations (2.5 and 3 at%, respectively) while N has a lower concentration of 1.7 at%. 

**Figure 5:**
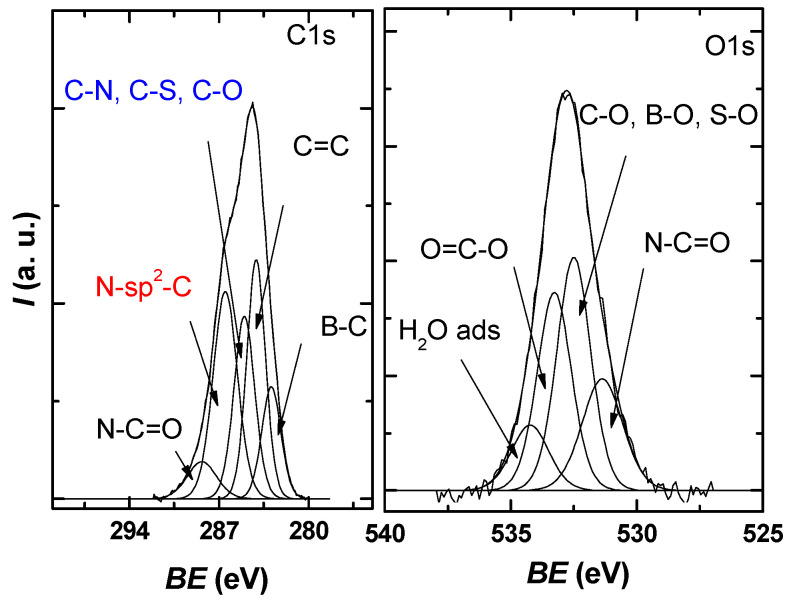
C1s and O1s XPS high-resolution spectra for the investigated sample, each with the deconvolution into characteristic components.

The obtained core level spectra O1s, C1s, S2p, B1s, and N1s confirmed the presence of the constituent elements in the graphene sample. The C1s high-resolution spectrum of the obtained sample (Figure 5) presents an asymmetrical, well-defined structure that can be deconvoluted into five distinct species, resulting in an FWHM average of 1.4–2 eV per peak, which, according to the literature, is within reasonable limits [35,36,37].

The identified components correspond to B–C bonds at 283 eV, sp^2^ graphite 284.2 eV, C–N, C–S, and C–O bonds at 285 eV, N-Sp2-C bonds at 286.5 eV, and N-C=O bonds at 288.4 eV [38,39,40,41].

From the O1s deconvolution, it can be seen that three main components are present around 531.3, 532.4, and 533.2 eV in the investigated sample, which, according to the literature, can be attributed to N-C=O, C-O/B-O/S-O, and O=C-O. A small contribution is present in the region of high values of the binding energy due to the adsorbed water molecules. 

**Figure 6:**
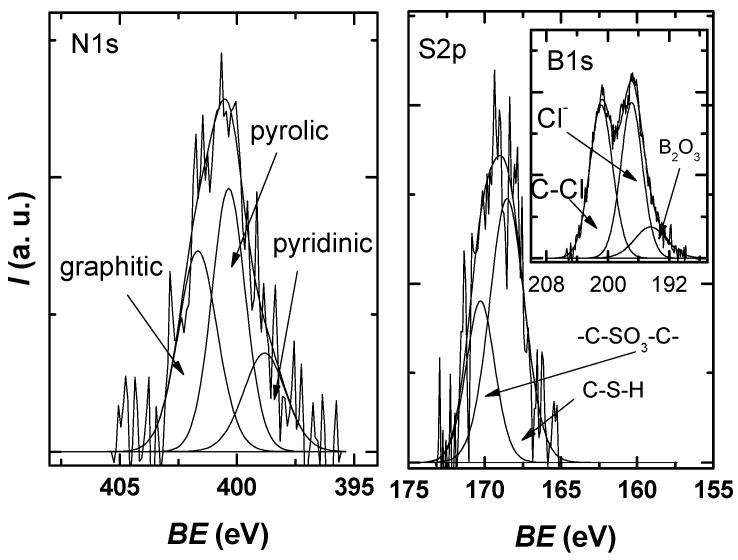
N1s and S2p XPS high-resolution spectra for the investigated sample, each with the deconvolution into characteristic components.

Figure 6 shows that the N1s spectra can be deconvoluted into three component peaks attributed to pyridinic-N (398.8 eV), pyrrolic-N (400.4 eV), and substitutional/graphitic-N (401.6 eV) [42,43]. Two sulfur species are observed in the S2p spectrum: the peak at 168.6 eV, which corresponds to C-S-H species, and the peak at 170.3 eV, which can be associated with -C-SO_3_-C- sulfate groups [44].

The high-resolution spectrum of B1s was deconvoluted using three components—the peak at 196.4 eV can be attributed to B_2_O_3_ species; the peak at 196.9 eV can be attributed to inorganic chlorine from a chloride salt with Na, K; the peak at 200 eV that is indicative of chlorine atoms covalently bonded to sp2 C—as reported for organochlorine compounds or due to Cl–C=O bonds [45,46]. 

### 3.2. Electrochemical Studies

**Scheme 2:**
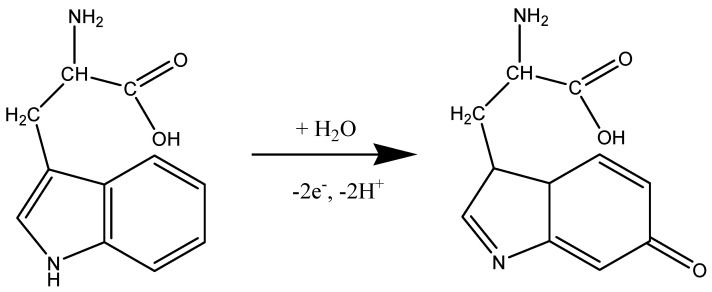
The suggested mechanism for electrochemical oxidation of L-tryptophan.

**Figure 7:**
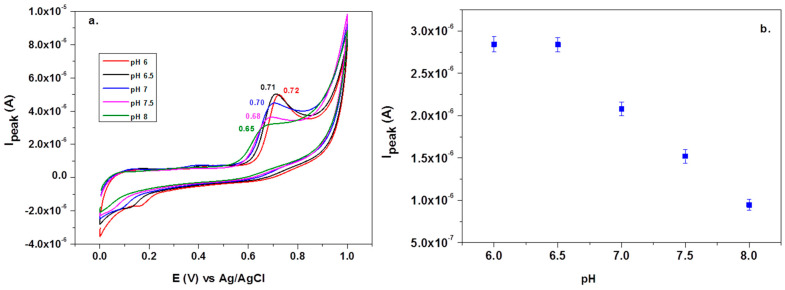
Cyclic voltammetry (CV) recorded with graphene-modified electrodes in solutions containing 10^−4^ M L-tryptophan in PBS solutions of various pH: 6, 6.5, 7, 7.5, and 8 (**a**). Variations of the peak current (I_peak_) with the solution pH (**b**).

**Figure 8:**
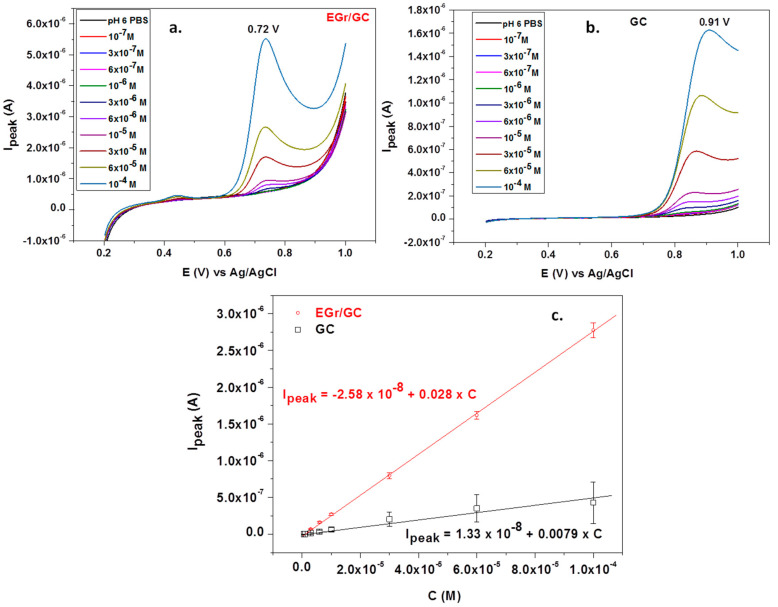
Linear sweep voltammograms recorded with a graphene-modified electrode (**a**) and a bare GC electrode (**b**) in the presence of different concentrations of L-tryptophan (supporting electrolyte: pH 6 PBS; scan rate: 10 mV/s); the corresponding calibration plots for bare GC and EGr/GC electrodes (**c**).

Before testing the electrocatalytic properties of the graphene-modified electrode towards L-tryptophan oxidation, the influence of the solution pH was studied. Figure 7a shows the CV response of 10^−4^ M L-tryptophan in PBS solutions of various pH (from 6 to 8), recorded with an EGr/GC electrode (scan rate: 10 mV/s). A well-defined oxidation peak can be observed at the potential of +0.72 V (pH 6 PBS) that slightly shifts to lower potentials with the increase of pH (e.g., +0.65 V in pH 8 PBS). A small reduction peak is present in the reverse scan, at +0.19 V, which can be attributed to the reduction of the oxidized intermediate of tryptophan [47]. In Figure 7b, one can see that the oxidation peak has similar values in slightly acidic solutions (2.9 × 10^−6^ A) but it strongly decreased at higher pH values. Based on the above results, the next experiments were recorded in pH 6 PBS solutions. Considering the results from the present study and the previous literature data [20,48], the possible electrochemical oxidation mechanism for L-tryptophan is as follows (see Scheme 2):

Under the optimized conditions, L-tryptophan was quantitatively analyzed by linear sweep voltammetry; the corresponding recordings at various concentrations (1 × 10^−7^–1 × 10^−4^ M) can be seen in Figure 8a,b. Significant differences are noticed between the bare and graphene-modified electrodes. In the first place, the peak potential is very high for the bare GC electrode (+0.91 V) compared with that of the graphene-modified electrode (+0.72 V). In addition, the peak intensity is three times larger for the EGr/GC electrode, confirming that the graphene layers considerably increase the transfer of electrons across the electrode/solution interface. 

The corresponding calibration curves obtained by plotting I_peak_ versus L-tryptophan concentrations are represented in Figure 8c. The oxidation peak current linearly increased with the concentration, and the corresponding regression equation was I_peak_ = 1.33 × 10^−8^ + 0.0078 × C, R^2^ = 0.9781 (for the bare GC electrode) and I_peak_ = −2.58 × 10^−8^ + 0.028 × C, R^2^ = 0.9978 (for the EGr/GC electrode). As expected, the EGr/GC electrode had high sensitivity (0.028 A/M) and a low limit of detection (3.03 × 10^−7^ M). The detection limit was calculated from the limit of determination (LOD) divided by 3.3. LOD is the lowest concentration of L-tryptophan for the electrode to give an oxidation signal.

According to several studies, the oxidation products of L-tryptophan can be easily adsorbed onto the electrode’s surface, fouling the electrical signal. Therefore, we tested the reproducibility of the signals given by the bare GC and EGr/GC electrodes in three successive measurements. After each measurement, the electrode was immersed in distilled water for 10 min, and then 10 voltammetric scans were recorded between the +0.2…+1 V potential range (10 mV/s; pH 6 PBS). The results can be seen in Supporting Information Material (Figure S1a,b) indicating very weak adsorption of the oxidation products on the EGr/GC electrode in comparison with the bare GC electrode. The relative standard deviation (RSD %) in a 10^−4^ M L-tryptophan solution was 3.72% for EGr/GC and 65.9% for GC. The results further confirmed the advantages of using the EGr material for electrode modification. 

**Figure 9:**
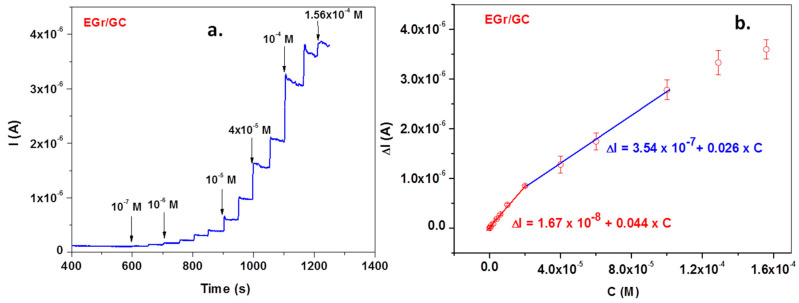
Amperometric curve recorded with the EGr/GC electrode in pH 6 PBS-supporting electrolyte (at +0.72 V) after adding various concentrations of L-tryptophan (TRP) (**a**). The corresponding calibration plot, showing t wo linear ranges (**b**).

Excellent signals were also obtained when the EGr/GC electrode was polarized at a potential of +0.72 V and various concentrations of L-tryptophan were added to the supporting electrolyte, pH 6 PBS (Figure 9a—amperometric measurement). The corresponding calibration curve obtained after subtracting the background current is represented in Figure 9b. In this case, the calibration plot has two linear ranges: the first one from 1 × 10^−7^ to 2 × 10^−5^ M and the second one from 2 × 10^−5^ to 10^−4^ M. Above 10^−4^ M, a saturation effect occurs and the curve deviates from linearity. Most important, in the low concentration range (1 × 10^−7^ to 2 × 10^−5^ M), electrode sensitivity is very high (0.044 A/M) and the limit of detection is even lower (3.03 × 10^−8^ M). 

**Figure 10:**
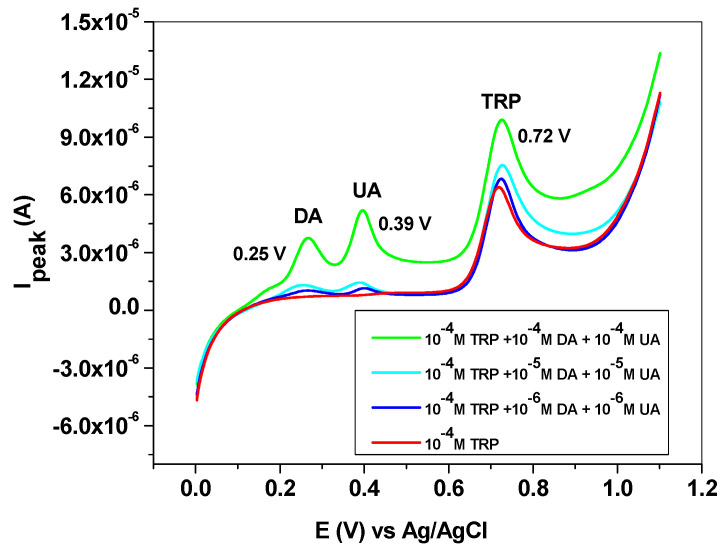
Linear sweep voltammetry (LSV) recorded with the EGr/GC electrode in the absence and presence of interfering species, dopamine, and uric acid. Scan rate: 10 mV/s; supporting electrolyte: pH 6 PBS.

The influence of some interfering species, such as dopamine (DA), uric acid (UA), ascorbic acid (AA) and melatonin (MEL), was next studied. DA and UA generally coexist with L-tryptophan in biological fluids; therefore, the study of their influence on TRP oxidation is highly important. Dopamine and uric acid can be electrochemically oxidized, and the corresponding peaks appear at potentials lower than that of L-tryptophan (Figure 10). Hence, dopamine gives a signal at +0.25 V, while uric acid oxidation takes place at +0.39 V. As expected, their influence on the L-tryptophan oxidation peak is closely related to their concentrations. This can be clearly seen in the LSVs recorded with the EGr/GC electrode in the presence and absence of interfering species. At low concentrations of interfering species (10^−6^ and 10^−5^ M), the peak value of TRP is slightly affected. At higher concentrations (10^−4^ M), a shift in the background current can be observed, accompanied by an increase of the TRP oxidation peak. In contrast, the peak potential of TRP (+0.72 V) did not change, regardless of DA and UA concentrations. 

**Figure 11:**
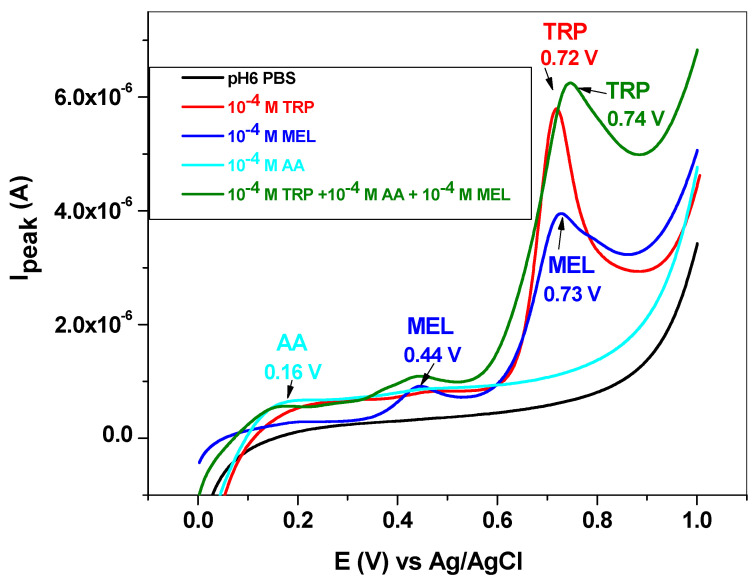
LSV recorded with the EGr/GC electrode in the absence and presence of interfering species, melatonin, and ascorbic acid. Scan rate: 10 mV/s; supporting electrolyte: pH 6 PBS.

Melatonin is a hormone produced in the tryptophan/serotonin pathway and has a great influence on the diurnal rhythm. It also influences the reproductive and immune systems and gastrointestinal motility. Its synthesis within the body is regulated by the blue light spectrum (e.g., 446 to 477 nm), and, during the night, it is secreted by the pineal gland, inducing neural and endocrine effects [14]. The electrochemical oxidation of melatonin takes place in two steps, giving rise to two broad peaks at +0.44 and +0.73 V (blue line, Figure 11). The second peak is very close to that corresponding to L-tryptophan (+0.72 V); therefore, it may significantly influence TRP oxidation.

Ascorbic acid or vitamin C has the role of cofactor in many enzymatic reactions that mediate several biological functions in the human body. It also acts as a reducing agent (antioxidant), and its deficiency in the body leads to impaired collagen synthesis. The oxidation of ascorbic acid takes place at low potential (+0.16 V), and the peak is weak and very broad (cyan line, Figure 11).

When mixed with L-tryptophan, the two interfering species (10^−4^ M for each of them) induced the increase of the capacitive current and also shifted the TRP peak potential from 0.72 to 0.74 V (see the olive curve in comparison with the red curve). Concerning the peak current of L-tryptophan, its value is lower in the presence of interfering species (2.7 × 10^−6^ versus 3.02 × 10^−6^ A), indicating that the antioxidant properties of ascorbic acid may prevent TRP oxidation.

**Figure 12:**
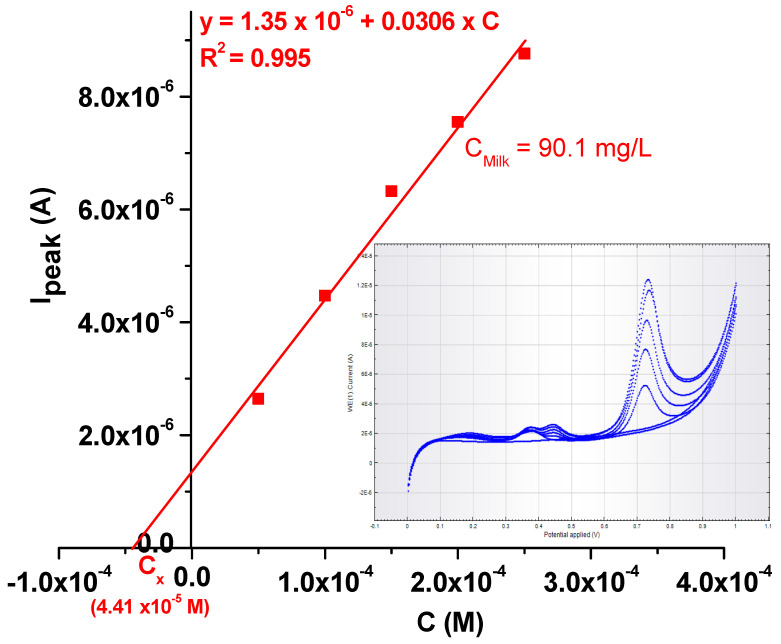
Calibration plot obtained with the EGr/GC electrode after standard addition of TRP in a milk sample. Inset: the LSVs recorded at various L-tryptophan concentrations (from 5 × 10^−5^ to 2.5 × 10^−4^ M) in milk.

In order to test the practical applicability of the EGr/GC-modified electrode, it was used to detect the L-tryptophan concentration in milk that was bought from the local supermarket (4% fat). Before measurements, 1 mL of milk was diluted 10-times with pH 6 PBS; then, increasing concentrations of TRP (also dissolved in pH 6 PBS) were added (see Figure 12). In the inset are presented the LSV curves recorded in the milk sample containing increasing concentrations of TRP (from 5 × 10^−5^ to 2.5 × 10^−4^ M). The final concentration of L-tryptophan in milk was determined by standard addition, and the value was found to be 90.1 mg/L. The results prove that the EGr/GC-modified electrode is sensitive for L-tryptophan determination in real samples.

The performances of the EGr/GC electrode were compared with those of other modified electrodes reported in the literature (see Table 3). Here, one can see that the electrode has an excellent performance in terms of both linear range and limit of detection.

## 4. Conclusions

A triple-doped (N, S, B) graphene sample was prepared by electrochemical exfoliation of graphite rods in a solution containing 0.05 M (NH_4_)_2_SO_4_ + 0.1 M H_3_BO_3_ + 0.05 M NaCl. After morphological and structural characterization of the graphene sample, its electrocatalytic properties towards L-tryptophan oxidation were studied. Significant differences were noticed between the bare GC and graphene-modified electrodes. In the first place, the TRP peak potential was very high for the bare electrode (+0.91 V) in comparison with that of the EGr/GC electrode (+0.72 V). Second, the peak intensity was three times larger for the EGr/GC electrode, confirming that the graphene layers considerably increased the transfer of electrons across the electrode/solution interface. In order to test the practical applicability of the EGr/GC-modified electrode, this was used to detect the L-tryptophan concentration in milk (4% fat). Its concentration was determined by standard addition, and the value was found to be 90.1 mg/L, proving that the electrode was sensitive enough for real sample analysis.

## Figures and Tables

**Table 1:** Structural parameters obtained from the corresponding XRD pattern: graphene crystallite size (*D*), interlayer distance *(d*), average number of layers (*n*), and their amount (*%*) within the graphene sample.

Sample	*2θ* (deg)	*D* (nm)	*d* (nm)	*n*	Amount
					(%)
EGr	9.66 (GO)	2.48	0.92	∼3	17
	21.68 (FLG)	1.18	0.41	∼3	69
	26.22 (MLG)	10.1	0.34	∼30	14

**Table 2:** Elemental concentrations of C, N, S, O, and B in the EGr sample.

Sample	Elemental Concentration				
EGr	C	N	S	O	B
	70.3%	1.7%	2.5%	22.4%	3%

**Table 3:** Comparison of the analytical performance of various electrodes modified with graphene-based materials.

Electrode/Sensing Material	Linear Range (M)	DL (M)	Ref.
carbon paste electrode/nitrogen-doped graphene nanosheets/CuCo_2_O_4_ nanoparticles	0.010–3.0 × 10^−6^	0.0041 × 10^−6^	[21]
GC/poly(L-methionine) and graphene composite film	0.2–150 × 10^−6^	0.017 × 10^−6^	[22]
GC/reduced graphene oxide and SnO_2_	1–100 × 10^−6^	0.04 × 10^−6^	[23]
GC/Cu_2_O-nanoparticle-coated reduced graphene oxide	0.02–20 × 10^−6^	0.01 × 10^−6^	[24]
GC/nitrogen-doped ordered mesoporous carbon	0.5–70.0 × 10^−6^ 70.0–200.0 × 10^−6^	3.5 × 10^−8^	[49]
GC/Co_3_O_4_ nanoparticles-decorated graphene	0.05–10 × 10^−6^	0.01 × 10^−6^	[50]
GC/nitrogen-, sulfur-, and boron-doped graphene	1 × 10^−7^–2 × 10^−5^	3.03 × 10^−8^	This work

## Data Availability

Data will be provided upon reasonable request to the corresponding author.

## Supplementary Materials

The following are available online at https://www.mdpi.com/2079-6374/11/2/36/s1, Figure S1: Calibration plots obtained from 3 successive measurements recorded with EGr/GC (a) and GC (b) electrodes.
