# Supplementary material for: Investigation of L-Tryptophan Electrochemical Oxidation with a Graphene-Modified Electrode
Source: Biosensors (Basel). 2021 Jan 28;11(2):36. doi: 10.3390/bios11020036 (PMC7911164; doi:10.3390/bios11020036)
Supplement: Supplementary file 1 [file biosensors-11-00036-s001.pdf]

Supporting Information

Investigation of L-Tryptophan electrochemical oxidation with graphene-modified electrode

Florina Pogacean, Codruta Varodi, Maria Coros, Irina Kacso, Teodora Radu, Bogdan Ionut Cozar, Valentin Mirel, and Stela Pruneanu*

National Institute for Research and Development of Isotopic and Molecular Technologies, Donat Street, no. 67-103, 400293 Cluj-Napoca, Romania

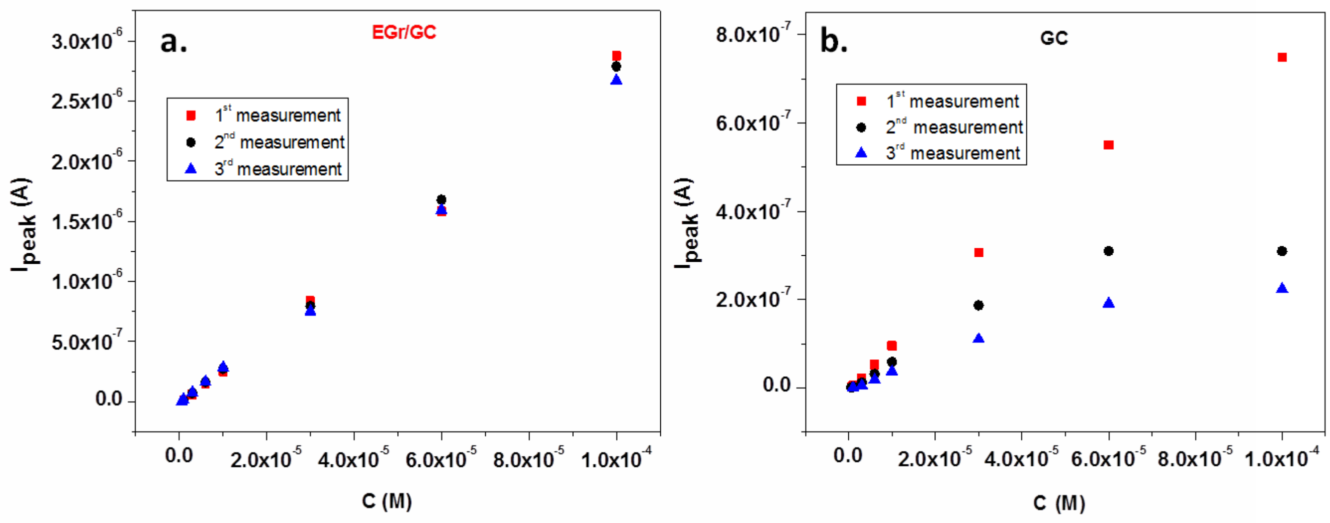

Figure S1. Calibration plots obtained from 3 successive measurements recorded with EGr/GC (a) and GC (b) electrodes.
